# Computational identification of key genes that may regulate gene expression reprogramming in Alzheimer’s patients

**DOI:** 10.1371/journal.pone.0222921

**Published:** 2019-09-23

**Authors:** Judith A. Potashkin, Virginie Bottero, Jose A. Santiago, James P. Quinn

**Affiliations:** 1 The Cellular and Molecular Pharmacology Department, The Chicago Medical School, Rosalind Franklin University of Medicine and Science, North Chicago, IL, United States of America; 2 NeuroHub Analytics, LLC, Chicago, IL, United States of America; 3 Q Regulating Systems, LLC, Gurnee, IL, United States of America; Nathan S Kline Institute, UNITED STATES

## Abstract

The dementia epidemic is likely to expand worldwide as the aging population continues to grow. A better understanding of the molecular mechanisms that lead to dementia is expected to reveal potentially modifiable risk factors that could contribute to the development of prevention strategies. Alzheimer’s disease is the most prevalent form of dementia. Currently we only partially understand some of the pathophysiological mechanisms that lead to development of the disease in aging individuals. In this study, Switch Miner software was used to identify key switch genes in the brain whose expression may lead to the development of Alzheimer’s disease. The results indicate that switch genes are enriched in pathways involved in the proteasome, oxidative phosphorylation, Parkinson’s disease, Huntington’s disease, Alzheimer’s disease and metabolism in the hippocampus and posterior cingulate cortex. Network analysis identified the krupel like factor 9 (KLF9), potassium channel tetramerization domain 2 (KCTD2), Sp1 transcription factor (SP1) and chromodomain helicase DNA binding protein 1 (CHD1) as key transcriptional regulators of switch genes in the brain of AD patients. These transcriptions factors have been implicated in conditions associated with Alzheimer’s disease, including diabetes, glucocorticoid signaling, stroke, and sleep disorders. The specific pathways affected reveal potential modifiable risk factors by lifestyle changes.

## Introduction

Dementia affects over 50 million people worldwide, approximately 67% of which have Alzheimer’s disease (AD) [1]. By 2050 it is predicted that as many as 152 million people may have dementia [1]. Unfortunately, there remains no cure for AD and only four drugs have been approved for treatment that manages symptoms of dementia in some patients. We currently do not completely understand the cause of AD, but there is strong data to support the involvement of the proteins amyloid and tau. In AD patients, amyloid-ß and hyperphosphorylated tau are produced abundantly in the brain. Amyloid-ß forms inter-neuronal plaques that disrupt cell function, whereas tau forms intra-neuronal neurofibrillary tangles that block intracellular transport. Risk factors for AD include genetics (i.e. *APOE epsilon 4* carrier), biology (i.e. aging and gender), and environmental factors (i.e. glucose and cholesterol metabolism, inflammation and oxidative stress) related to lifestyle choices or accidents (i.e. diet, exercise, smoking, education and head trauma).

Plaques and tangles may appear in the brain 18 years before the onset of symptoms [2]. Initially plaques and tangles form in the hippocampus (HIP) and entorhinal cortex (EC) of the temporal lobe, which are involved in learning and memory [3]. In addition to the HIP and EC, metabolic and pathological differences have been found in the middle temporal gyrus (MTG), posterior cingulate cortex (PCC), and superior frontal gyrus (SFG) of AD patients [4–24]. The primary visual cortex (VCX) usually does not show disease-related neurodegeneration [25, 26]. Recently, laser capture microdissection (LCM) was used to select neurons in regions of the brain affected in AD patients and healthy elderly controls. Gene expression profiling of the neurons identified changes that occur in the development and pathogenesis of AD [19, 26]. One striking finding from these studies is that AD patients have significantly reduced expression of mitochondrial transport chain genes in the PCC, MTG and HIP compared to controls [27].

The key events in AD development remain unknown, but gene expression studies on postmortem brain tissue are expected to reveal pathways that are dysregulated in patients. Recently SWItch Miner (SWIM) software has been developed that combines gene expression data with topological properties of correlation networks to reveal major changes in cellular phenotype that are at the root of biological processes [28]. In SWIM, the Pearson correlation coefficient between the expression of two genes is used to build co-expression networks. RNA transcripts are the nodes of the network and connections between nodes are made if the expression of the genes is significantly correlated or anti-correlated. Clustering algorithms are used to identify disease modules. SWIM analysis identifies “switch genes” that may be fundamental to a disease. SWIM has been used to identify switch genes involved in the transformation of glioblastomas from stem-like to the differentiated state [29] and reprogramming in grapevine development from immature to mature growth [30]. The use of SWIM could be expanded to the study of chronic diseases in order to reveal key players that lead to disease development.

In this study, we have used SWIM to identify genes whose expression is associated with drastic changes in the brain of AD patients. The results show that the switch genes in the HIP and PCC regions of the brain that are affected in AD patients are enriched in proteasome, oxidative phosphorylation, metabolic, Parkinson’s disease, Huntington’s disease, and Alzheimer’s disease pathways.

## Methods

### Database mining

**Fig 1:**
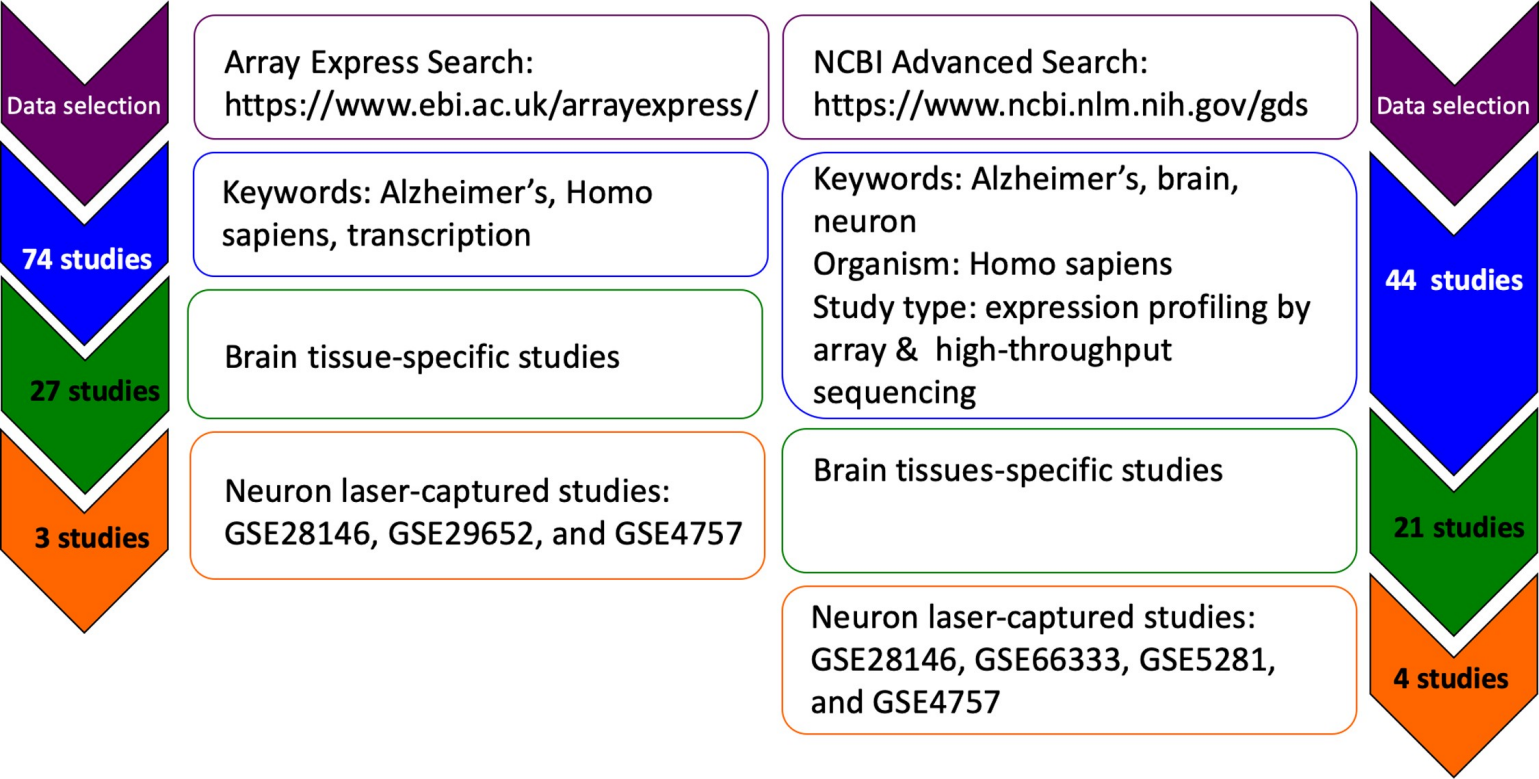
Database mining. The NCBI GEO database (https://www.ncbi.nlm.nih.gov/gds) and ArrayExpress database (https://www.ebi.ac.uk/arrayexpress/) were searched on June 3, 2019 for studies in which gene expression data was available from laser-captured neurons in the brain of Alzheimer’s patients.

The NCBI GEO database (https://www.ncbi.nlm.nih.gov/gds) and ArrayExpress database (https://www.ebi.ac.uk/arrayexpress/) were searched on June 3, 2019 for studies in which gene expression data was available from laser-captured neurons in the brain of Alzheimer’s patients (Fig 1). The NCBI GEO database was queried using the search terms Alzheimer’s, brain, neuron and "Homo sapiens"[Organism]) for the study types expression profiling by array and expression profiling by high-throughput sequencing. 44 studies were identified, 21 were brain-specific studies and 4 had data from laser-captured neurons (GSE28146, GSE66333, GSE5281, GSE4757). The ArrayExpress database was searched using the keywords Alzheimer’s, Homo sapiens, and transcription and 74 studies were identified, 27 of these studies were brain-specific and 3 studies had data from laser-captured neurons (GSE28146, GSE29652, and GSE4757).

### SWIM analysis to identify switch genes

**Fig 2:**
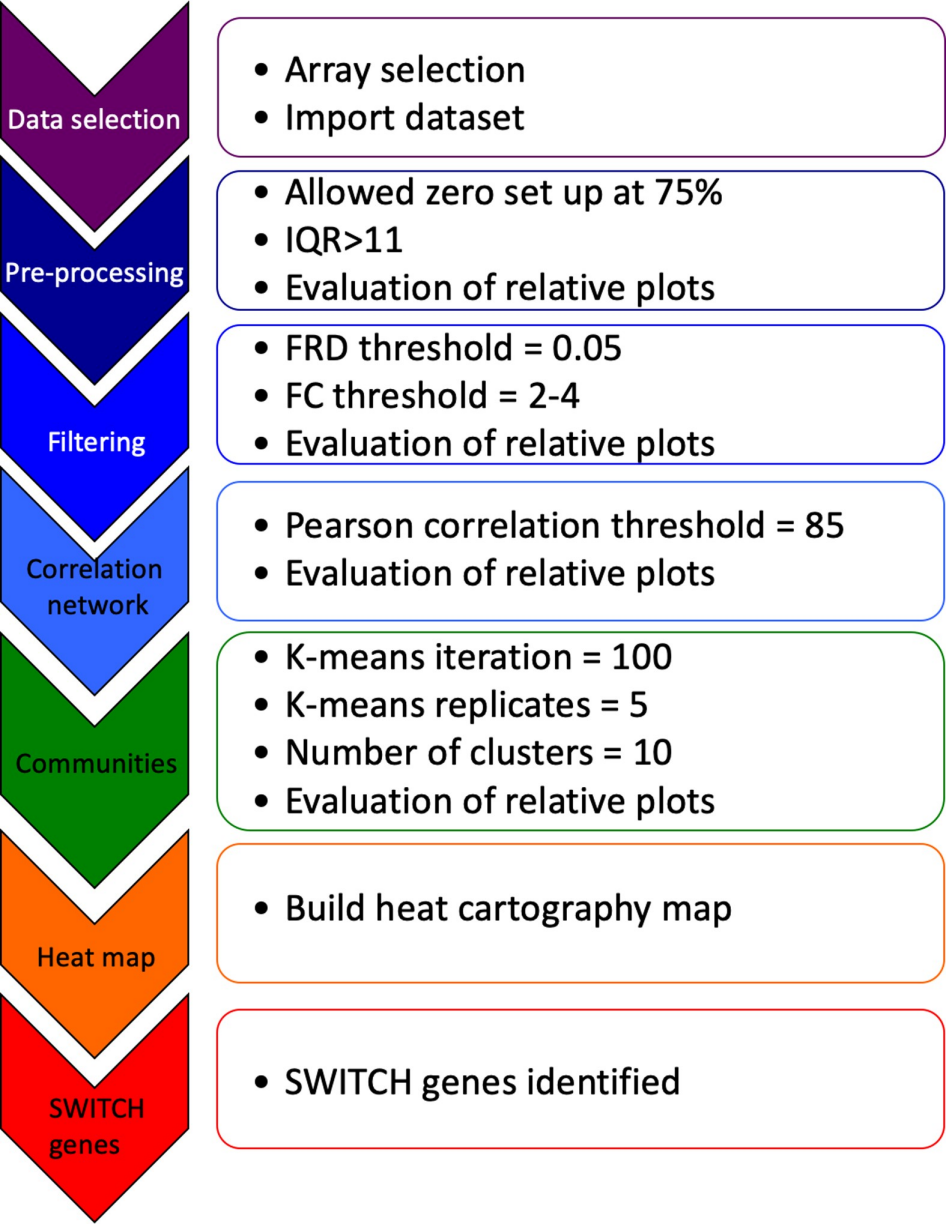
SWIM analysis to identify switch genes. Data from the expression arrays was imported into SWIM. The SWIM algorithm is comprised of the steps depicted in the figure [31]. For each brain section and each group, diseased and normal, the mean value of the gene expression is calculated for every cell in the microarray. The p-value is then calculated for each gene. Control for the false discovery rate is performed using the Benjamini and Hochberg approach. The sets of genes to analyze is further filtered by setting thresholds for the magnitude of the fold change and false discovery rate. Next, the Pearson correlation coefficient is calculated for the remaining pairs of genes and those below a threshold are eliminated. At this point 1000 to 2000 genes out of the original 54675 remain. These genes become the nodes in the network that will be analyzed.

Raw data from the expression arrays was imported into SWIM. The SWIM algorithm is comprised of several steps shown in Fig 2 [31]. In the pre-processing phase, genes that are not expressed or only slightly expressed are removed. In the filtering phase, the fold-change limit was set between 2–4 and genes that were not significantly expressed differently between AD patients compared to controls are removed. The False Discovery Rate method was used to correct for multiple tests [32] and then a Pearson correlation analysis was used to build a co-expression network of genes differentially expressed between AD patients and controls. In step 4, the k-means algorithm was then used to identify communities within the network [33]. To determine the number of clusters, SWIM uses Scree plot, which allows replicating the clustering many times with a new set of initial cluster centroid positions, and for each replicate the k-means algorithm performs iterations until the minimum of the sum of the squared error (SSE) function is reached. The cluster configuration with the lowest SSE values among the replicates is designated as the number of clusters. The heat cartography map is built using a clusterphobic coefficient *Kπ*, which measures external and internal node connections, and the global within-module degree *Zg*, which measures the extent each node is connected to others in its own community. When *Zg* exceeds 5 a node it is considered a hub. The average Pearson correlation coefficient (APCC) between the expression profiles of each node and its nearest neighbors is used to build the heat cartography map. Using APCC, three types of hubs may be identified. Date hubs show low positive co-expression with their partners (low APCC), party hubs show high positive co-expression (high APCC), and nodes that have negative APCC values are called fight-club hubs [28]. In the final step of SWIM analysis, switch genes are identified that are a subset of the fight-club hubs that interact outside of their community. Switch genes are characterized as not being a hub in their own cluster (low *Zg* <2.5), having many links outside their own cluster (*Kπ* >0.8, when *Kπ* is close to 1 most of its links are external to its own module), and having a negative average weight of incident links (APCC <0) [28].

The switch genes are the set of genes that interact outside their own community, are not in local hubs and are mainly anti-correlated with their interaction partners. After the switch genes are identified, KEGG pathway analysis was conducted to see if the results imply a relationship to a disease. The random set of genes is chosen from the identified nodes from above. The analysis is performed by studying the effect on the network connectivity of removing different types of nodes by decreasing degree. The total number of nodes to be removed must be equal to the total number of switch genes and the cumulative node deletion is carried out by type (i.e., total hubs, party hubs, date hubs, fight-club hubs, switch genes, and randomly chosen nodes).

### Pathway analysis

Entrez gene identifiers from the SWIM analysis were imported into the Database for Annotation, Visualization and Integrated Discovery (DAVID) (https://david.ncifcrf.gov/) which uses singular enrichment analysis [34, 35]. The functional annotation tool was used to select Kyoto Encyclopedia of Genes and Genomes (KEGG) pathway analysis. The biological functions of KEGG charts were enriched with *p* < .05.

### Transcription factor analysis

Entrez gene identifiers from the SWIM analysis were imported into NetworkAnalyst for network analysis of transcription factors [36]. In NetworkAnalyst, we used the transcription factor and gene target data derived from the ENCODE ChIP-seq data. Transcription factor analysis in NetworkAnalyst uses the BETA Minus Algorithm in which only peak intensity signal < 500 and the predicted regulatory potential score <1 is used. Transcription factors were ranked according to network topology measurements including degree and betweenness centrality.

## Results

In order to identify gene expression changes in the brain that may lead to the transition from a healthy aging brain to that of AD patient, we used the SWIM algorithm. The Array Express and NCBI databases were searched to identify studies that contained expression data from postmortem brain tissue of AD patients and age-matched controls (Fig 1). LCM neuron studies (GSE28146, GSE29652, GSE4757, GSE66333 and GSE5281) and several microarrays with samples from brain sections were identified. SWIM analysis was conducted on each of these datasets. In all of the brain section studies and four of the LCM studies, the p values were not sufficiently robust to complete SWIM analysis. Only GSE5281 had data with sufficiently robust p values for the analysis to be completed.

The characteristics of the participants in the GSE5281 study [19, 26, 27] are presented in Table 1. Brain samples from a total of 22–23 participants (10 AD patients and 12–13 controls, depending on the brain region). The average age of the participants was 83 years old. The controls were matched as closely as possible for age at death and mean education level. The AD patients had a Braak stage ranging from III-IV [26].

**Table 1:** Characteristics of study participants.

GSE5281	Entorhinal	cortex	
Variable	HC	AD	*P* Value
Number of participant	13	10	
Age, mean (SD)	80.3 (9.2)	85.6 (6.3)	0.13
Female/male, No (% male)	3/10 (76)	6/4 (40)	0.07
GSE5281	Hippocampus		
Variable	HC	AD	*P* Value
Number of participant	13	10	
Age, mean (SD)	79.6 (9.4)	77.8 (5.7)	0.6
Female/male, No (% male)	3/10 (76)	4/6 (60)	0.38
GSE5281	Mid	temporal	gyrus
Variable	HC	AD	*P* Value
Number of participant	12	10	
Age, mean (SD)	80.1 (9.8)	79.1 (6.4)	0.76
Female/male, No (% male)	4/8 (67)	6/10 (63)	0.82
GSE5281	Posterior	cingulate	cortex
Variable	HC	AD	*P* Value
Number of participant	13	10	
Age, mean (SD)	79.8 (9.4)	77.5 (6.2)	0.56
Female/male, No (% male)	4/9 (69)	3/6 (67)	0.90
GSE5281	Superior	frontal	gyrus
Variable	HC	AD	*P* Value
Number of participant	13	10	
Age, mean (SD)	79.3 (10.2)	79.2 (7.5)	0.97
Female/male, No (% male)	4/7 (64)	10/13 (67)	0.69
GSE5281	Primary	visual	cortex
Variable	HC	AD	*P* Value
Number of participant	13	10	
Age, mean (SD)	79.9 (7)	80.2 (6.7)	0.38
Female/male, No (% male)	3/9 (75)	8/11 (58)	0.33

**Fig 3:**
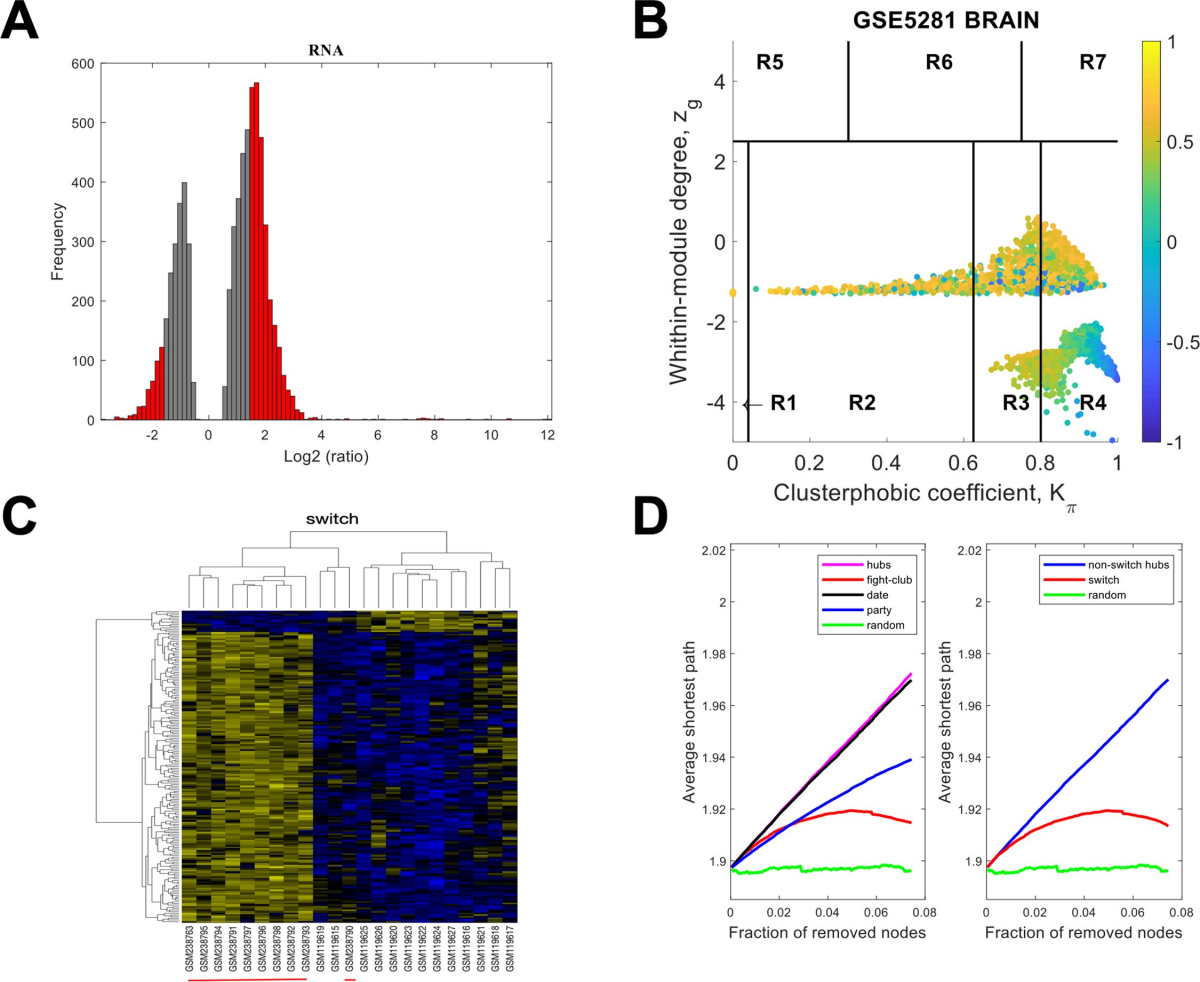
SWIM analysis of the entorhinal cortex. **A.** Distribution of the fold-change values for GSE5281 brain microarray gene expression data from the EC. The x-axis represents the fold-change value (log2 of the fold-change) that is the ratio of the average expression data in AD patients compared to the average expression data in normal controls computed for protein-coding and non-coding RNAs. The y-axis represents the frequency of the obtained fold-change values. The grey bars represent the fold-change values associated with protein-coding and non-coding RNAs that will be discarded according to the selected threshold. The red bars represent the fold-change values associated with protein-coding and non-coding RNAs that were retained for further analysis. **B.** Heat cartography map for GSE5281 brain data from the entorhinal cortex correlation network. The plane is identified by two parameters: *Zg* (within-module degree) and *Kπ* (clusterphobic coefficient) and it is divided into seven regions each defining a specific node role (R1-R7). High *Zg* values correspond to nodes that are hubs within their module (local hubs), whereas low *Zg* values correspond to nodes with few connections within their module (non-hubs within their communities, but they could be hubs in the network). Each node is colored according to its average Pearson Correlation coefficient (APCC) value. Yellow nodes are party and date hubs, which are positively correlated in expression with their interaction partners. Blue nodes are the fight-club hubs, which have an average negative correlation in expression with their interaction partners. Blue nodes falling in the region R4 are the switch genes, which are characterized by low *Zg* and by high *Kπ* values and are connected mainly outside their module. **C.** Dendrogram and heat map for switch genes in GSE5281 brain microarray gene expression data from the entorhinal cortex. The expression profiles of switch genes (including protein-coding and non-coding RNAs) are clustered according to rows (switch genes) and columns (samples) of the switch genes expression data (biclustering). The colors represent different expression levels that increase from blue to yellow. The red line under the x axis labels denotes AD samples. **D.** Robustness for the GSE5281 brain correlation network from the entorhinal cortex. The x-axis represents the cumulative fraction of removed nodes, while the y-axis represents the average shortest path. The shortest path between two nodes is the minimum number of consecutive edges connecting them. Each curve corresponds to the variation of the average shortest path of the correlation network as function of the removal of nodes specified by the colors of each curve.

Gene expression data from laser-captured neurons from HIP, EC, MTG, PCC, SFG and VCX was imported into SWIM. The data for EC using a linear fold-change of 3 is presented in Fig 3. The samples that were filtered out in step 2 of the analysis are depicted as grey bars in Fig 3A, whereas those that are retained for further analysis are shown in red. In Fig 3B the correlation communities are identified. The fight-club hubs are depicted in R4 in blue and are negatively correlated in expression with their interaction partner. A heat map of the expression of the switch genes, step 5, is shown in Fig 3C. After the switch genes are identified, the data is analyzed further to assess robustness. The data in Fig 3D indicates that fight-club hubs differ from date and party hubs and the switch genes are significantly different that random. The switch genes identified in the EC are listed in S1 Table.

**Fig 4:**
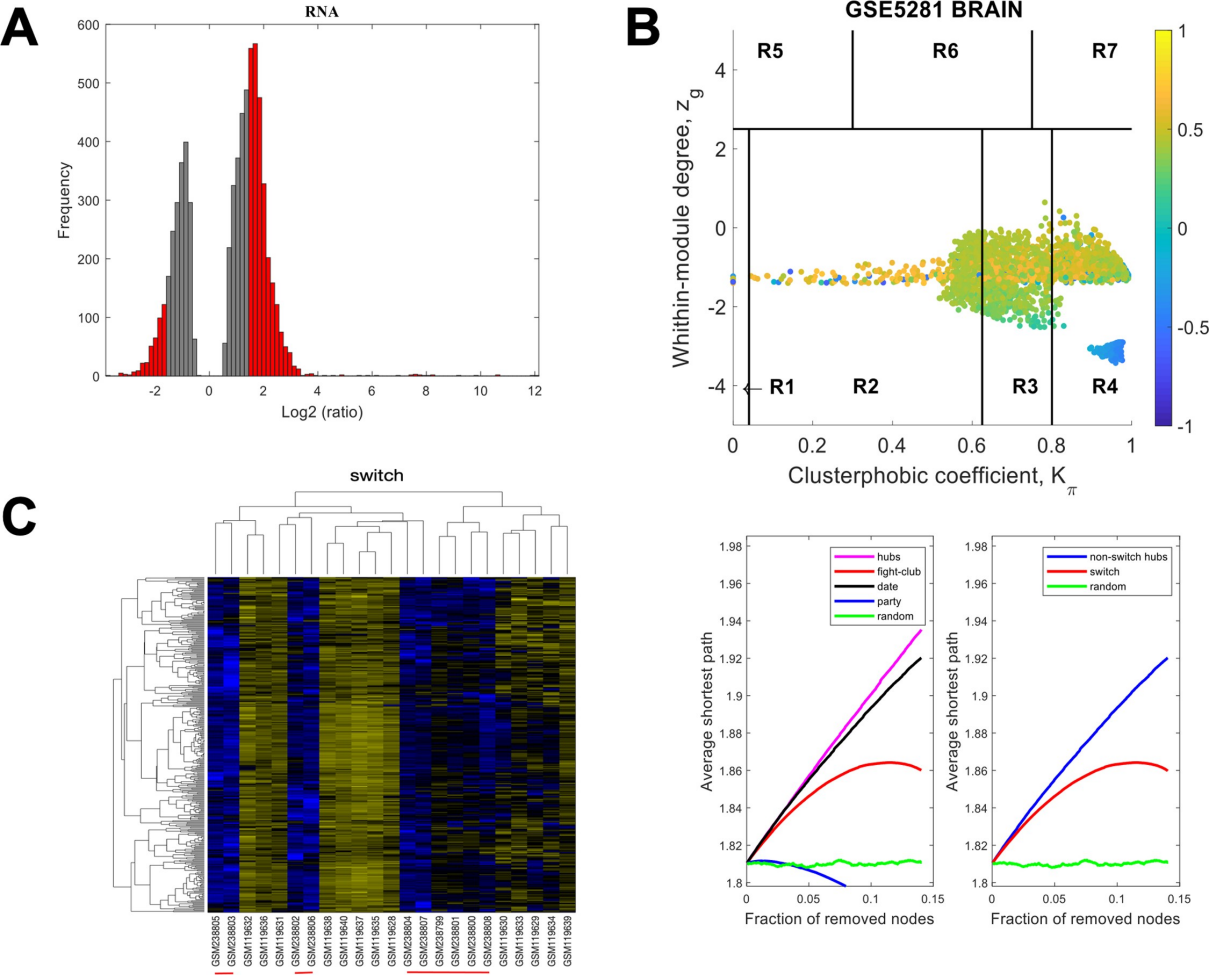
SWIM analysis of the hippocampus. **A.** Distribution of the fold-change values for GSE5281 brain microarray gene expression data from the HIP. The x-axis represents the fold-change value (log2 of the fold-change) that is the ratio of the average expression data in AD patients compared to the average expression data in normal controls computed for protein-coding and non-coding RNAs. The y-axis represents the frequency of the obtained fold-change values. The grey bars represent the fold-change values associated with protein-coding and non-coding RNAs that will be discarded according to the selected threshold. The red bars represent the fold-change values associated with protein-coding and non-coding RNAs that were retained for further analysis. **B.** Heat cartography map for GSE5281 brain data from the HIP. correlation network. The plane is identified by two parameters: *Zg* (within-module degree) and *Kπ* (clusterphobic coefficient) and it is divided into seven regions each defining a specific node role (R1-R7). High *Zg* values correspond to nodes that are hubs within their module (local hubs), whereas low *Zg* values correspond to nodes with few connections within their module (non-hubs within their communities, but they could be hubs in the network). Each node is colored according to its average Pearson Correlation coefficient (APCC) value. Yellow nodes are party and date hubs, which are positively correlated in expression with their interaction partners. Blue nodes are the fight-club hubs, which have an average negative correlation in expression with their interaction partners. Blue nodes falling in the region R4 are the switch genes, which are characterized by low *Zg* and by high *Kπ* values and are connected mainly outside their module. **C.** Dendrogram and heat map for switch genes in GSE5281 brain microarray gene expression data from the HIP. The expression profiles of switch genes (including protein-coding and non-coding RNAs) are clustered according to rows (switch genes) and columns (samples) of the switch genes expression data (biclustering). The colors represent different expression levels that increase from blue to yellow. The red line under the x axis labels denotes AD samples. **D.** Robustness for the GSE5281 brain correlation network from the HIP. The x-axis represents the cumulative fraction of removed nodes, while the y-axis represents the average shortest path. The shortest path between two nodes is the minimum number of consecutive edges connecting them. Each curve corresponds to the variation of the average shortest path of the correlation network as function of the removal of nodes specified by the colors of each curve.

The data for HIP using a linear fold-change of 3 is presented in Fig 4. The samples that are retained for further analysis are depicted in red in Fig 4A, the correlation communities are identified in Fig 4B and the fight-club hubs are depicted in R4 in blue. A heat map of the expression of the switch genes, is shown in Fig 4C. The data indicates that fight-club hubs differ from date and party hubs and the switch genes are significantly different that random, Fig 4D. The switch genes identified in the HIP are listed in S2 Table.

**Fig 5:**
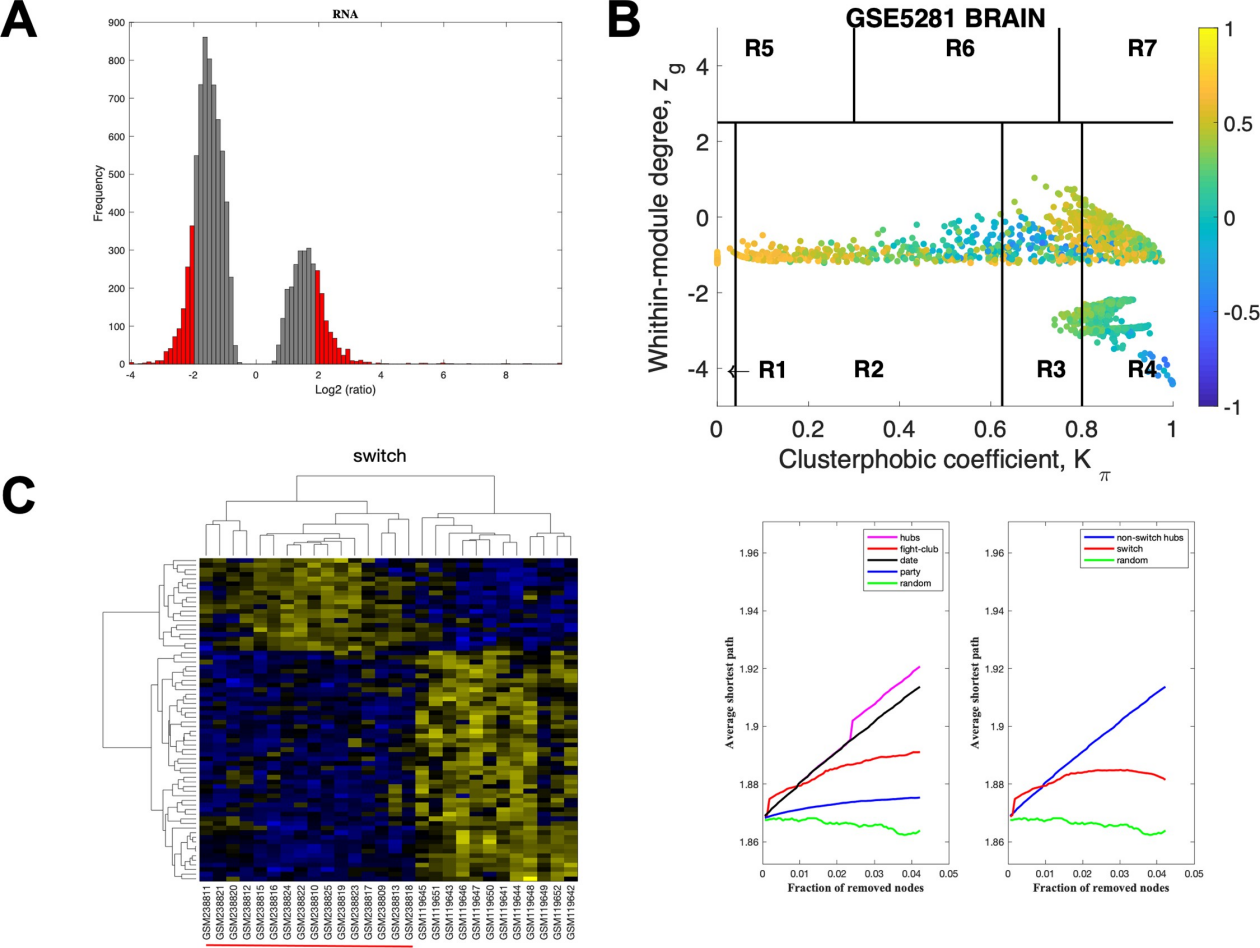
SWIM analysis of the mid temporal gyrus. **A.** Distribution of the fold-change values for GSE5281 brain microarray gene expression data from the MTG. The x-axis represents the fold-change value (log2 of the fold-change) that is the ratio of the average expression data in AD patients compared to the average expression data in normal controls computed for protein-coding and non-coding RNAs. The y-axis represents the frequency of the obtained fold-change values. The grey bars represent the fold-change values associated with protein-coding and non-coding RNAs that will be discarded according to the selected threshold. The red bars represent the fold-change values associated with protein-coding and non-coding RNAs that were retained for further analysis. **B.** Heat cartography map for GSE5281 brain data from the MTG. correlation network. The plane is identified by two parameters: *Zg* (within-module degree) and *Kπ* (clusterphobic coefficient) and it is divided into seven regions each defining a specific node role (R1-R7). High *Zg* values correspond to nodes that are hubs within their module (local hubs), whereas low *Zg* values correspond to nodes with few connections within their module (non-hubs within their communities, but they could be hubs in the network). Each node is colored according to its average Pearson Correlation coefficient (APCC) value. Yellow nodes are party and date hubs, which are positively correlated in expression with their interaction partners. Blue nodes are the fight-club hubs, which have an average negative correlation in expression with their interaction partners. Blue nodes falling in the region R4 are the switch genes, which are characterized by low *Zg* and by high *Kπ* values and are connected mainly outside their module. **C.** Dendrogram and heat map for switch genes in GSE5281 brain microarray gene expression data from the MTG. The expression profiles of switch genes (including protein-coding and non-coding RNAs) are clustered according to rows (switch genes) and columns (samples) of the switch genes expression data (biclustering). The colors represent different expression levels that increase from blue to yellow. The red line under the x axis labels denotes AD samples. **D.** Robustness for the GSE5281 brain correlation network from the MTG. The x-axis represents the cumulative fraction of removed nodes, while the y-axis represents the average shortest path. The shortest path between two nodes is the minimum number of consecutive edges connecting them. Each curve corresponds to the variation of the average shortest path of the correlation network as function of the removal of nodes specified by the colors of each curve.

The data for MTG using a linear fold-change of 4 is presented in Fig 5. The samples that are retained are depicted in red, Fig 5A and the correlation communities are identified in Fig 5B with the fight-club hubs depicted in R4 in blue. A heat map of the expression of the switch genes is presented in Fig 5C. The data indicates that fight-club hubs differ from date and party hubs and the switch genes are significantly different that random, Fig 5D. The switch genes identified in the MTG are listed in S3 Table.

**Fig 6:**
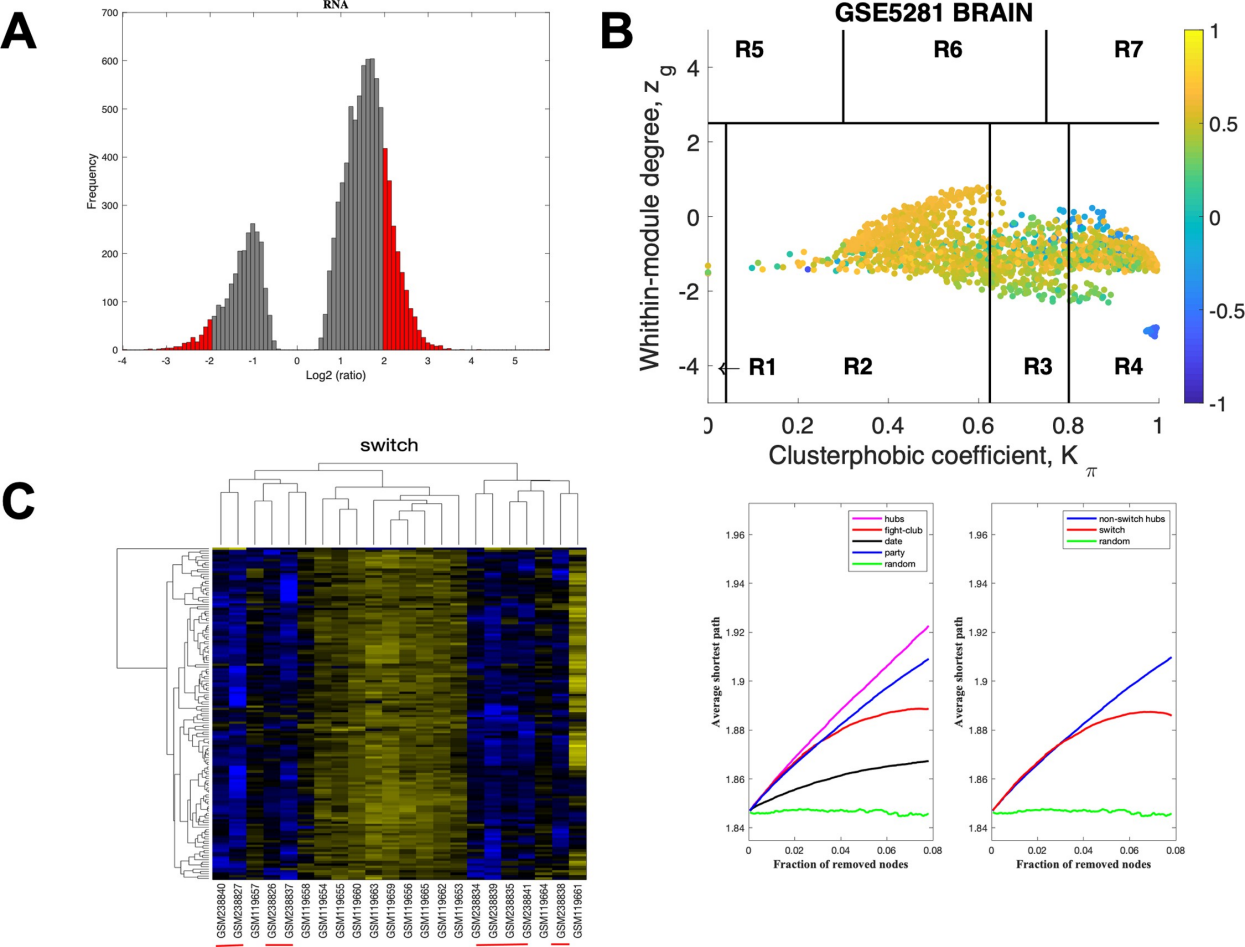
SWIM analysis of the posterior cingulate. **A.** Distribution of the fold-change values for GSE5281 brain microarray gene expression data from the PC. The x-axis represents the fold-change value (log2 of the fold-change) that is the ratio of the average expression data in AD patients compared to the average expression data in normal controls computed for protein-coding and non-coding RNAs. The y-axis represents the frequency of the obtained fold-change values. The grey bars represent the fold-change values associated with protein-coding and non-coding RNAs that will be discarded according to the selected threshold. The red bars represent the fold-change values associated with protein-coding and non-coding RNAs that were retained for further analysis. **B.** Heat cartography map for GSE5281 brain data from the PC. correlation network. The plane is identified by two parameters: *Zg* (within-module degree) and *Kπ* (clusterphobic coefficient) and it is divided into seven regions each defining a specific node role (R1-R7). High *Zg* values correspond to nodes that are hubs within their module (local hubs), whereas low *Zg* values correspond to nodes with few connections within their module (non-hubs within their communities, but they could be hubs in the network). Each node is colored according to its average Pearson Correlation coefficient (APCC) value. Yellow nodes are party and date hubs, which are positively correlated in expression with their interaction partners. Blue nodes are the fight-club hubs, which have an average negative correlation in expression with their interaction partners. Blue nodes falling in the region R4 are the switch genes, which are characterized by low *Zg* and by high *Kπ* values and are connected mainly outside their module. **C.** Dendrogram and heat map for switch genes in GSE5281 brain microarray gene expression data from the PC. The expression profiles of switch genes (including protein-coding and non-coding RNAs) are clustered according to rows (switch genes) and columns (samples) of the switch genes expression data (biclustering). The colors represent different expression levels that increase from blue to yellow. The red line under the x axis labels denotes AD samples. **D.** Robustness for the GSE5281 brain correlation network from the PC. The x-axis represents the cumulative fraction of removed nodes, while the y-axis represents the average shortest path. The shortest path between two nodes is the minimum number of consecutive edges connecting them. Each curve corresponds to the variation of the average shortest path of the correlation network as function of the removal of nodes specified by the colors of each curve.

The data for PCC using a linear fold-change of 4 is presented in Fig 6. The samples that are retained for further analysis are depicted in red, Fig 6A, the correlation communities are identified in Fig 6B with the fight-club hubs depicted in R4 in blue. A heat map of the expression of the switch genes, step 5, is shown in Fig 6C. The data indicates that fight-club hubs differ from date and party hubs and the switch genes are significantly different that random, Fig 6D. The switch genes identified in the PCC are listed in S4 Table.

**Fig 7:**
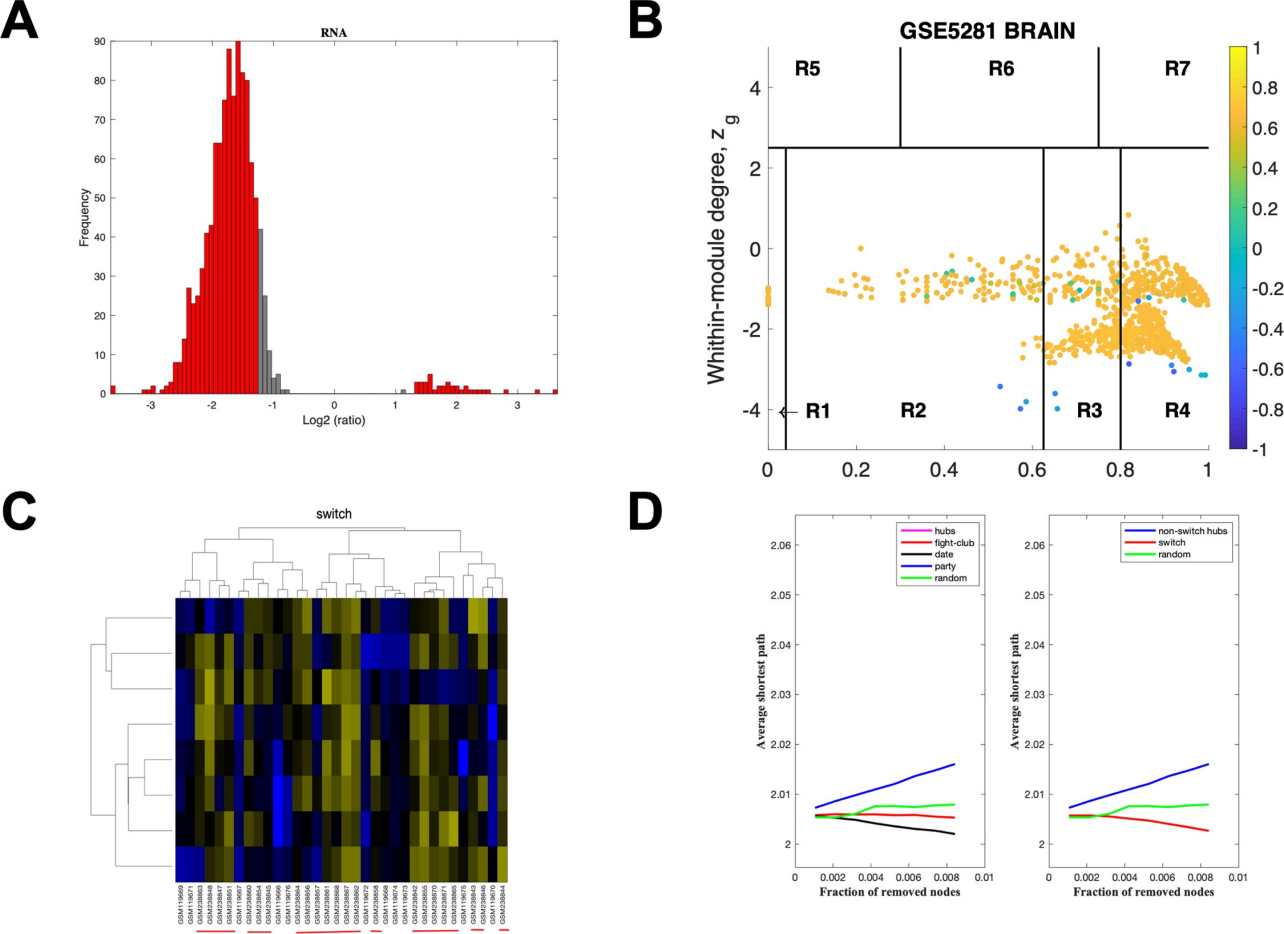
SWIM analysis of the superior frontal gyrus. **A.** Distribution of the fold-change values for GSE5281 brain microarray gene expression data from the SFG. The x-axis represents the fold-change value (log2 of the fold-change) that is the ratio of the average expression data in AD patients compared to the average expression data in normal controls computed for protein-coding and non-coding RNAs. The y-axis represents the frequency of the obtained fold-change values. The grey bars represent the fold-change values associated with protein-coding and non-coding RNAs that will be discarded according to the selected threshold. The red bars represent the fold-change values associated with protein-coding and non-coding RNAs that were retained for further analysis. **B.** Heat cartography map for GSE5281 brain data from the SFG. correlation network. The plane is identified by two parameters: *Zg* (within-module degree) and *Kπ* (clusterphobic coefficient) and it is divided into seven regions each defining a specific node role (R1-R7). High *Zg* values correspond to nodes that are hubs within their module (local hubs), whereas low *Zg* values correspond to nodes with few connections within their module (non-hubs within their communities, but they could be hubs in the network). Each node is colored according to its average Pearson Correlation coefficient (APCC) value. Yellow nodes are party and date hubs, which are positively correlated in expression with their interaction partners. Blue nodes are the fight-club hubs, which have an average negative correlation in expression with their interaction partners. Blue nodes falling in the region R4 are the switch genes, which are characterized by low *Zg* and by high *Kπ* values and are connected mainly outside their module. **C.** Dendrogram and heat map for switch genes in GSE5281 brain microarray gene expression data from the SFG. The expression profiles of switch genes (including protein-coding and non-coding RNAs) are clustered according to rows (switch genes) and columns (samples) of the switch genes expression data (biclustering). The colors represent different expression levels that increase from blue to yellow. The red line under the x axis labels denotes AD samples. **D.** Robustness for the GSE5281 brain correlation network from the SFG. The x-axis represents the cumulative fraction of removed nodes, while the y-axis represents the average shortest path. The shortest path between two nodes is the minimum number of consecutive edges connecting them. Each curve corresponds to the variation of the average shortest path of the correlation network as function of the removal of nodes specified by the colors of each curve.

For SFG, initially the fold-change was set at 3 and SWIM was not able to identify any switch genes. We then set the linear fold-change of 2.5 and the data obtained from SWIM analysis is presented in Fig 7. The samples that are retained for further analysis are depicted in red, Fig 7A, the correlation communities are identified in Fig 7B and very few fight-club hubs were found. A heat map of the expression of the switch genes, step 5, is shown in Fig 7C. The data indicates that fight-club hubs differ from date and party hubs, but the switch genes differ only slightly from random, Fig 7D. The switch genes identified in the SFG are listed in S5 Table.

**Fig 8:**
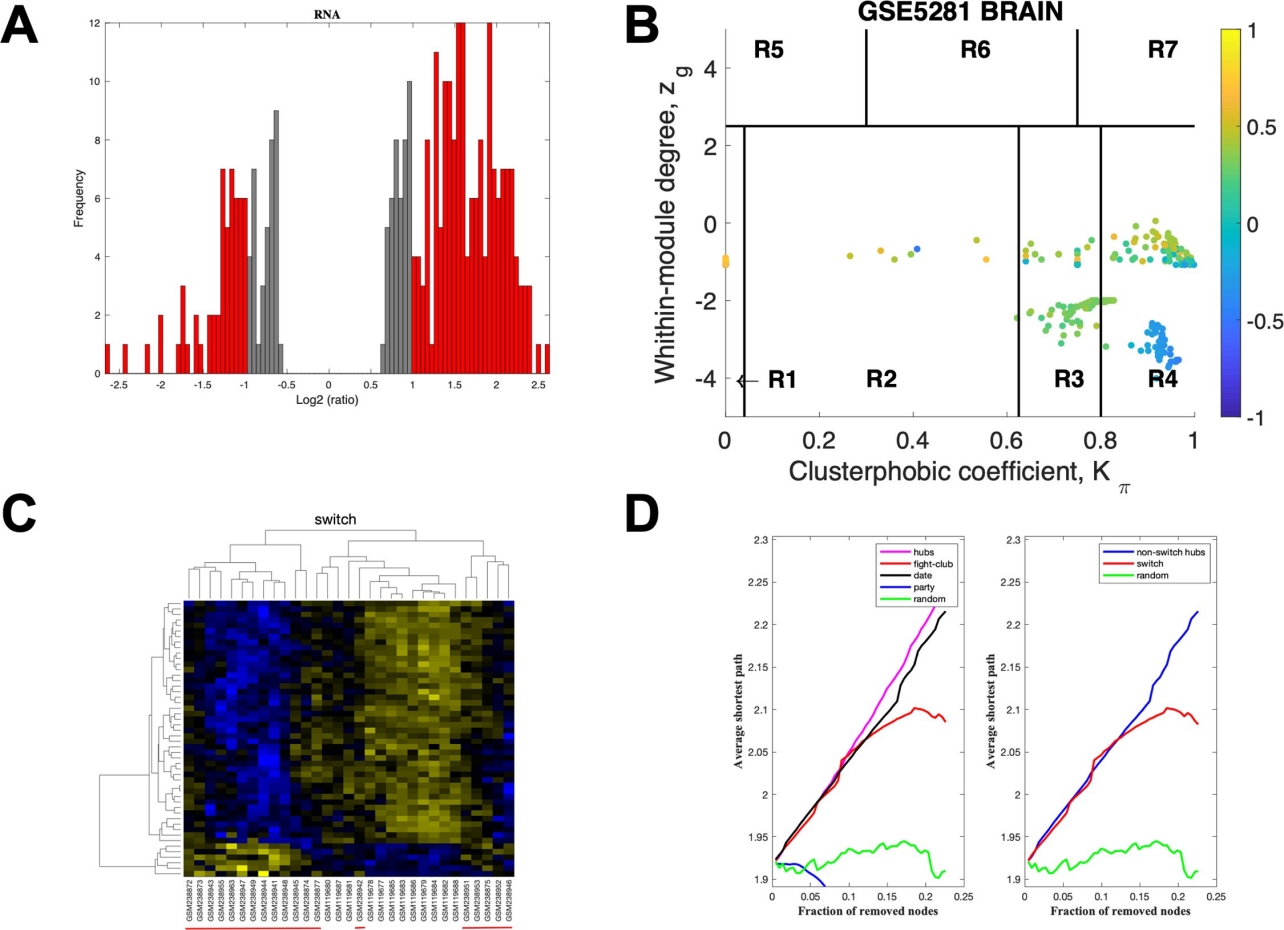
SWIM analysis of the primary visual cortex. **A.** Distribution of the fold-change values for GSE5281 brain microarray gene expression data from the VCX. The x-axis represents the fold-change value (log2 of the fold-change) that is the ratio of the average expression data in AD patients compared to the average expression data in normal controls computed for protein-coding and non-coding RNAs. The y-axis represents the frequency of the obtained fold-change values. The grey bars represent the fold-change values associated with protein-coding and non-coding RNAs that will be discarded according to the selected threshold. The red bars represent the fold-change values associated with protein-coding and non-coding RNAs that were retained for further analysis. **B.** Heat cartography map for GSE5281 brain data from the VCX. correlation network. The plane is identified by two parameters: *Zg* (within-module degree) and *Kπ* (clusterphobic coefficient) and it is divided into seven regions each defining a specific node role (R1-R7). High *Zg* values correspond to nodes that are hubs within their module (local hubs), whereas low *Zg* values correspond to nodes with few connections within their module (non-hubs within their communities, but they could be hubs in the network). Each node is colored according to its average Pearson Correlation coefficient (APCC) value. Yellow nodes are party and date hubs, which are positively correlated in expression with their interaction partners. Blue nodes are the fight-club hubs, which have an average negative correlation in expression with their interaction partners. Blue nodes falling in the region R4 are the switch genes, which are characterized by low *Zg* and by high *Kπ* values and are connected mainly outside their module. **C.** Dendrogram and heat map for switch genes in GSE5281 brain microarray gene expression data from the VCX. The expression profiles of switch genes (including protein-coding and non-coding RNAs) are clustered according to rows (switch genes) and columns (samples) of the switch genes expression data (biclustering). The colors represent different expression levels that increase from blue to yellow. The red line under the x axis labels denotes AD samples. **D.** Robustness for the GSE5281 brain correlation network from the VCX. The x-axis represents the cumulative fraction of removed nodes, while the y-axis represents the average shortest path. The shortest path between two nodes is the minimum number of consecutive edges connecting them. Each curve corresponds to the variation of the average shortest path of the correlation network as function of the removal of nodes specified by the colors of each curve.

For VCX, initially the fold-change was set at 3 and SWIM was not able to identify any switch genes. We then set the linear fold-change at 2 and the data obtained from SWIM analysis is presented in Fig 8. The samples that are retained for further analysis are depicted in red in Fig 8A and the correlation communities are identified in Fig 8B. The fight-club hubs are depicted in R4 in blue. A heat map of the expression of the switch genes, step 5, is shown in Fig 8C. The data indicates that fight-club hubs do not differ measurably from date and party hubs, but the switch genes are significantly different than random, Fig 8D. The switch genes identified in the VCX are listed in S6 Table.

**Fig 9:**
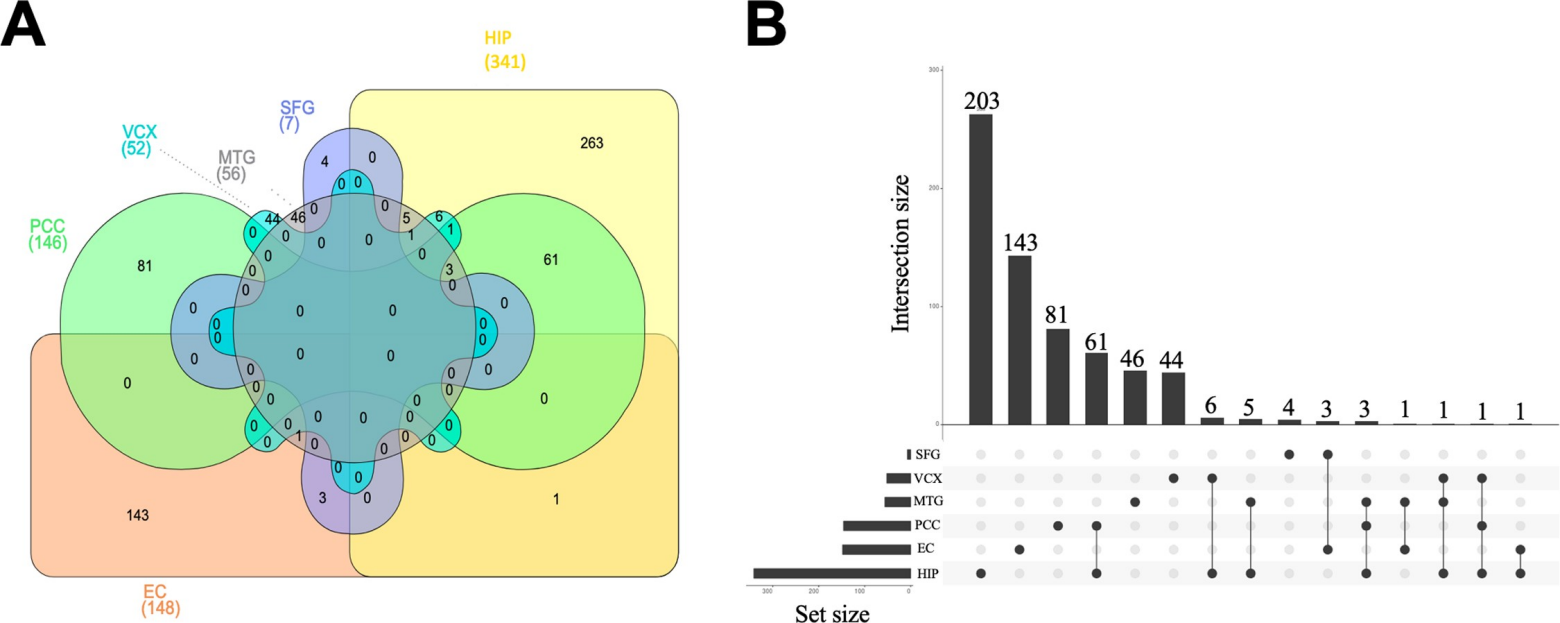
Venn diagram and UpsetR plot of switch genes from different brain regions. A. The Venn diagram was created using http://www.interactivenn.net/. The genes symbols were imported for the different brain areas. EC: Entorhino cortex, HIP: Hippocampus, MTG: Mid Temporal Gyrus, SFG: Superior Frontal Gyrus, PCC Posterior Cingulate, VCX: Primary Visual Cortex. B. The UpSetR plot was created as described [37]. The horizontal bars with labels at the lower left of the panel represent the six data sets that were included in the Venn diagram, with the length of each bar displaying the total set size. The dot pattern to the right shows the intersections between the sets. The vertical bars at the top show the size of the corresponding intersection, ranked by decreasing set size, where a gray dot indicates an empty set and a single black dot indicates no intersection with another set.

A Venn diagram analysis and UpSetR plot analysis of the switch genes identified in each brain region is shown in Fig 9 and S7 Table. The order of brain regions which have the largest number of switch genes to the least number is HIP>PCC>EC>MTG>VCX>SFG. The regions that had the largest number of unique switch genes to that which had the least is HIP>EC>PCC>MTG>VCX>SFG. The PCC shares 53 switch genes with the HIP (S7 Table). Interestingly, the EC shares only 1 switch gene with HIP and one with the PCC. Most of the EC switch genes are unique for that region of the brain.

**Fig 10:**
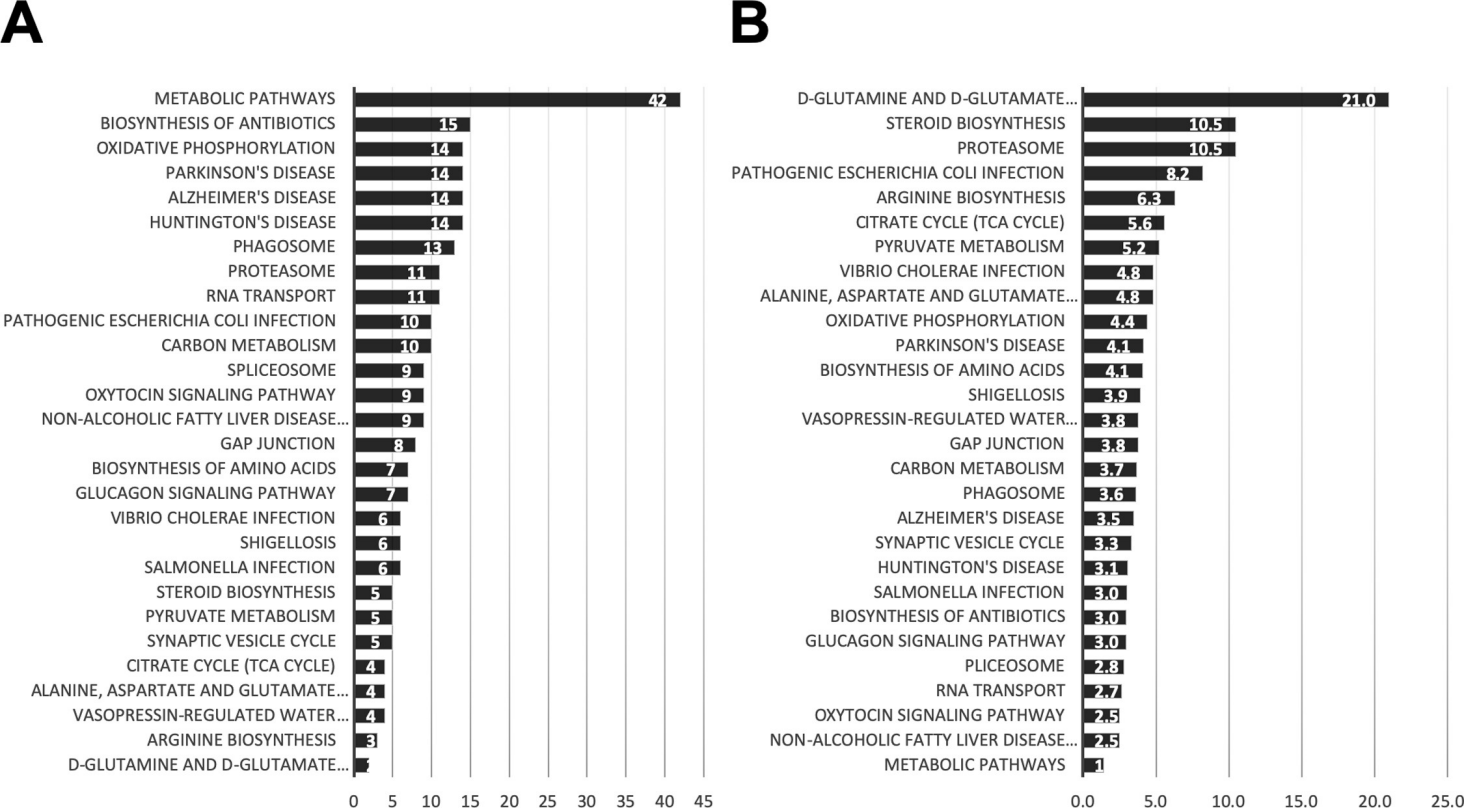
Hippocampus pathway enrichment analysis. Kyoto Encyclopedia of Genes and Genomes (KEGG) pathway enrichment analysis of swim genes of the HIP was performed using the Database for Annotation, Visualization and Integrated Discovery (DAVID). The gene count for each pathway is represented in A, whereas B represents the fold enrichment.

**Fig 11:**
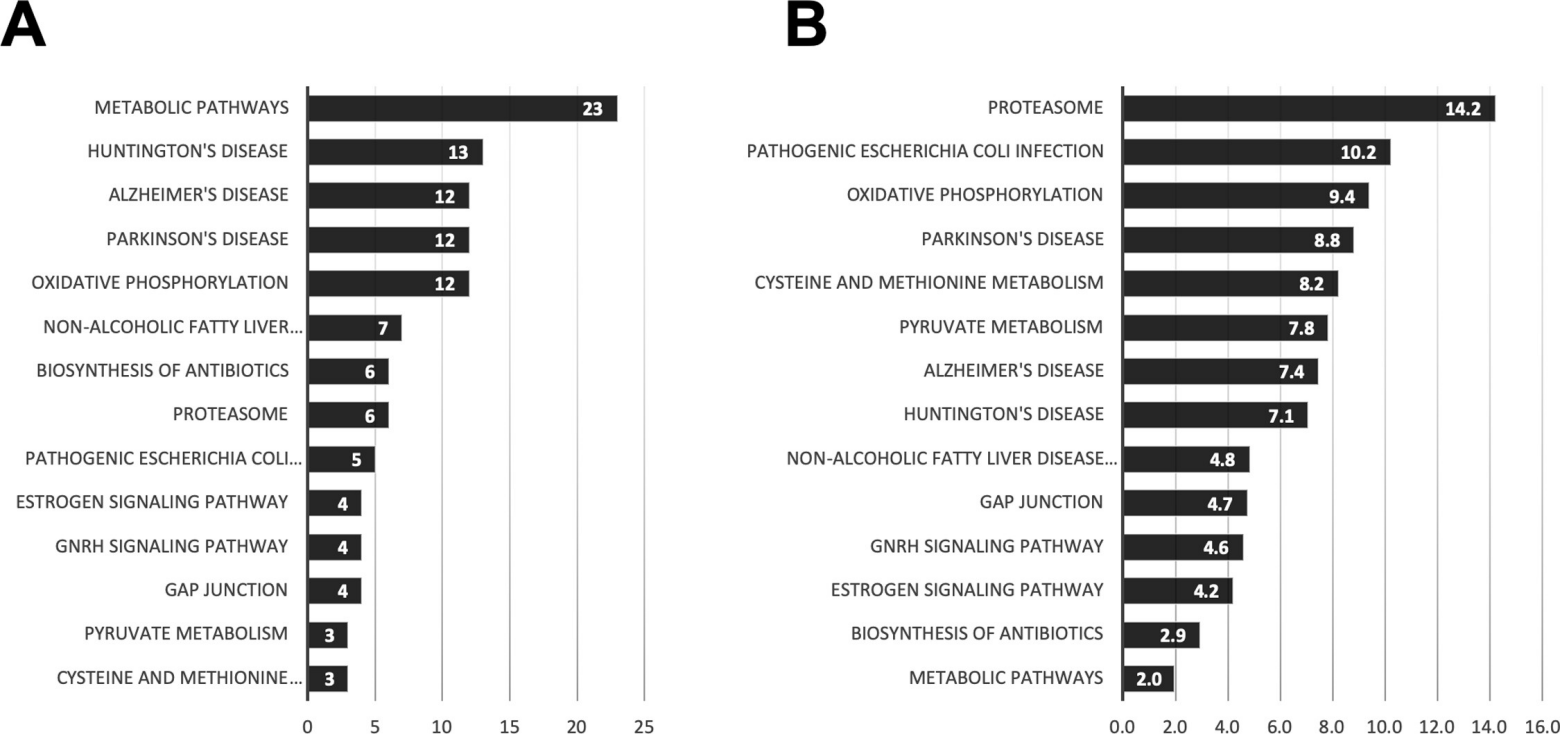
Posterior cingulate cortex (PCC) pathway enrichment analysis. KEGG pathway enrichment analysis of swim genes of the posterior cingulate cortex was performed using DAVID. The gene counts for each pathway is represented in A, whereas B represents the fold enrichment.

Pathway analysis of the switch genes was performed in order to identify functions. In the HIP the majority of the disrupted pathways were involved with metabolism, specifically glutamine, glutamate, steroid, arginine, pyruvate and amino acids metabolism (Fig 10). Changes in gene expression involved with oxidative phosphorylation, RNA transport and the spliceosome are enriched in switch genes. The switch genes of the HIP are also enriched in Parkinson’s, Alzheimer’s and Huntington’s disease pathways. The PCC switch genes are also enriched in metabolic and Parkinson’s, Alzheimer’s and Huntington’s disease pathways (Fig 11). The switch genes dysregulated pathways shared in the PCC and HIP are the proteasome, oxidative phosphorylation and metabolism (S1 Fig and S7 Table). The switch genes in the EC, MTG, VCX and SFG are not enriched in any particular pathways.

In order to identify key transcriptional regulators of the switch genes from the different brain regions, a transcription factor analysis was performed using NetworkAnalyst [36]. Network analysis was performed using the brain regions with the greater number of switch genes, the HIP and PCC. Network analysis revealed that switch genes identified in the HIP region were regulated by the transcription factors, krupel like factor 9 (KLF9), and potassium channel tetramerization domain 2 (KCTD2), whereas those from the PCC region were regulated by KLF9, Sp1 transcription factor (SP1), and chromodomain helicase DNA binding protein 1 (CHD1). As noted above, KLF9 was shared between the HIP and PCC brain regions (S3 and S4 Figs).

## Discussion

In this study we used SWIM analysis to identify key genes in regions of the brain known to show metabolic and pathological differences in AD patients compared to healthy aging individuals including the HIP, PCC, EC, MTG, and SFG. For comparison we also analyzed gene expression data from the VCX, which usually does not show disease-related neurodegeneration. Transcription data from laser-captured neurons was interrogated to identify switch genes. The results indicate that changes in gene expression in both the HIP and PCC may alter brain function by disrupting metabolism, oxidative phosphorylation, and the proteasome (S1 Fig).

Previous studies have shown that the PCC and HIP are affected in AD patients [12]. The PCC shows a reduction in glucose metabolism in early AD and has the largest abnormal positron emission tomography scans of cognitively normal late-middle age individuals who carry the *APOE epsilon 4* allele [24, 38, 39]. The HIP shows neurofibrillary tangles in AD patients [12, 14–16]. In addition, energy metabolism genes showed lower expression levels in the PCC and HIP in AD patients compared to controls [27].

In contrast to our study, a previous study that analyzed gene expression changes using the same microarray data found that cellular physiological processes, transport, metabolism and cellular localization were pathways affected across most brain regions including the PCC, HIP, EC, and MTG [26]. The unique aspect of the SWIM algorithm that most likely explains the difference in the results is that it includes fight-club hubs that are negatively correlated with their interaction partners. Therefore, although there is a dysregulation of gene expression that leads to pathways affected in multiple brain regions in AD patients, our data indicate that key switch gene changes that alter significant pathways are present mainly in in the PCC and HIP.

Our results also showed that switch genes in the EC, MTG, VCX and SFG are not enriched in any particular pathways. This suggests that these brain regions may be more resistant to key switch events that cause neurodegeneration compared to the PCC and HIP regions. Similar to our findings, the earlier study that analyzed the GSE5281 data found that the SFG and VCX areas, which are affected in later stages of AD, are relatively neuroprotected and capable of resisting disease pathology [26].

Caberlotto and colleagues used network analysis of AD-related genes to conclude that metabolism-associated processes including insulin and fatty acid metabolism underlie the development of AD [40]. In this study, seed genes associated with AD were obtained from the same transcriptomic data we used (microarray GSE5281). In addition, they used single nucleotide polymorphism data from AD, molecular targets of AD drugs and AD genes present in the Online Mendelian Inheritance in Man database. We compared the switch genes identified in our study to the AD-related seed genes identified by Caberlotto and colleagues and the results indicate that many of the switch genes were the same as the seed genes, especially in the HIP and PCC strongly suggesting that dysregulation of metabolic processes are key events important to the development of AD (S2 Fig and S8 Table).

Network analysis of the switch genes in the HIP and PCC brain regions identified several transcription factors relevant to the pathogenesis of AD. For example, network analysis identified KLF9 and KCTD2 as the main regulatory transcription factors of the HIP switch genes. Recently, Cui and colleagues demonstrated that KLF9 promotes the expression of peroxisome proliferator-activated receptor γ coactivator 1α (PGC1 α) resulting in hepatic gluconeogenesis and suggested that KLF9 may be responsible for the glucocorticoid therapy-induced diabetes [41]. In this context, diabetes has been extensively associated with an increased risk for AD [42]. Moreover, glucocorticoid overexposure has been associated with cognitive decline, amyloid beta misprocessing and ultimately, the development of AD [43, 44]. Given the involvement of KLF9 in glucose homeostasis and glucocorticoid signaling, its potential as a therapeutic target for AD warrants further investigation.

In addition to KLF9, KCTD2 was another key transcription factor regulating the HIP switch genes. Genome wide association studies identified KCTD2 as a shared susceptibility gene between AD and ischemic stroke [45, 46]. Interestingly, KCTD2 may play a role in sleep regulation [47, 48] and sleep disturbances have been linked to the development of AD [49].

Similarly, network analysis identified KLF9, SP1 and CHD1 as central regulators of PCC switch genes. Dysregulation of SP1 in AD has been documented in several studies. For instance, SP1 mRNA was upregulated in brains of both human and transgenic AD model mice [50]. Inhibition of SP1 function in a transgenic AD model mice increased memory deficits suggesting that it may be a useful therapeutic target [51]. Another important transcription factor, CHD1, is involved in TDP-43 mediated neurodegeneration [52]. Recently, Chd1 has been found to play a role in learning and memory in mice [53]. Furthermore, Chd1 knockdown in mouse embryonic stem cells mimicked high fat diet and aging-induced gene expression changes [54]. Collectively, the transcription factors identified in this study are involved in processes related to the pathogenesis of AD and thus may be important therapeutic targets.

There is a potential caveat that should be kept in mind when interpreting the results from this study. Although several possible gene expression datasets were identified, only one study achieved the high stringent p-values required for the SWIM analysis. Therefore, the results presented herein may be specific for this dataset and not of AD in general. Nonetheless, the pathways and transcription factors identified in this study have been associated with AD by other investigations. Future studies will seek to confirm the validity of these findings in an independent microarray.

## Conclusions

This study provides novel insights into the key switch events that occur in the HIP and PCC involved in the transformation from a healthy aging brain to that of an AD patient. The majority of the pathways in the HIP and PCC that are altered in AD patients are involved with metabolism including disruption of glutamine, glutamate, steroid, arginine, pyruvate and amino acids metabolism. In addition, some of the transcriptional regulators of the switch genes are involved in glucose homeostasis, glucocorticoid signaling, sleep regulation, and memory. Targeting these transcription factors may provide novel therapeutics for AD.

## Supporting information

S1 Fig Genes common in HIP and PCC pathway enrichment analysis. KEGG pathway enrichment analysis of swim genes common between the HIP and the PCC were performed using DAVID. The gene counts for each pathway is represented in A whereas B represent the fold enrichment.
(TIFF)

S2 Fig Venn diagram between SWIM genes and Caberlotto et al seed genes. The Venn diagrams were created using http://www.interactivenn.net/. The orange sets represent the SWIM genes whereas the green sets represent the seed genes from Caberlotto et al study.
(TIF)

S3 Fig Transcription factor analysis of the switch genes identified in the hippocampus. Network analysis of HIP switch genes was performed using NetworkAnalyst. Transcription factor data was derived from the ENCODE ChIP-seq database. Transcription factors (blue rectangles) and switch genes (pink circles) are ranked according to network topology measurements, degree and betweenness centrality. Transcription factors with the highest values of degree and betweenness centrality measurements are enclosed in the yellow oval. Gray lines represent protein-protein interactions. Network analysis was performed on June 2019.
(TIF)

S4 Fig Transcription factor analysis of the switch genes identified in the posterior cingulate cortex. Network analysis of posterior cingulate cortex switch genes was performed using NetworkAnalyst. Transcription factor data was derived from the ENCODE ChIP-seq database. Transcription factors (blue rectangles) and switch genes (pink circles) are ranked according to network topology measurements, degree and betweenness centrality. Transcription factors with the highest values of degree and betweenness centrality measurements are enclosed in the yellow oval. Gray lines represent protein-protein interactions. Network analysis was performed on June 2019.
(TIF)

S1 Table Enthorinal cortex SWIM genes. (XLSX)

S2 Table Hippocampus SWIM genes. (XLSX)

S3 Table Mid temporal gyrus SWIM genes. (XLSX)

S4 Table Posterior cingulate cortex SWIM genes. (XLSX)

S5 Table Superior frontal gyrus SWIM genes. (XLSX)

S6 Table Primary visual cortex SWIM genes. (XLSX)

S7 Table HIP and PCC SWIM genes venn diagram. (XLSX)

S8 Table Hippocampus pathway enrichment analysis. (XLSX)

S9 Table Posterior cingulate cortex pathway enrichment analysis. (XLSX)

S10 Table HIP/PCC pathway enrichment analysis. (XLSX)
