# Supplementary figures and images for: Computational identification of key genes that may regulate gene expression reprogramming in Alzheimer’s patients
Source: PLoS One. 2019 Sep 23;14(9):e0222921. doi: 10.1371/journal.pone.0222921 (PMC6756555; doi:10.1371/journal.pone.0222921)

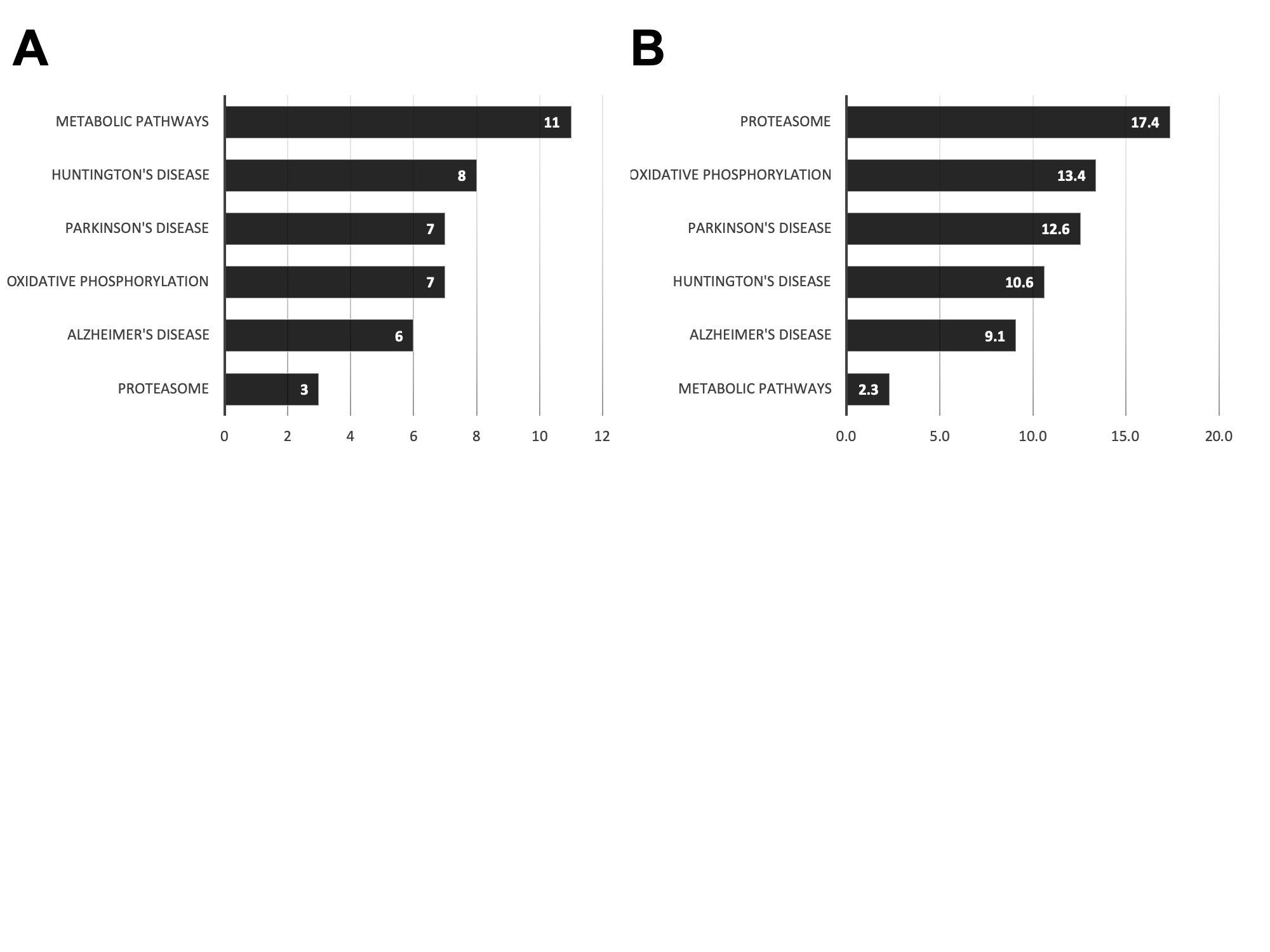

Supplement: S1 Fig — KEGG pathway enrichment analysis of swim genes common between the HIP and the PCC were performed using DAVID. The gene counts for each pathway is represented in A whereas B represent the fold enrichment. (TIFF) [file pone.0222921.s001.tiff]

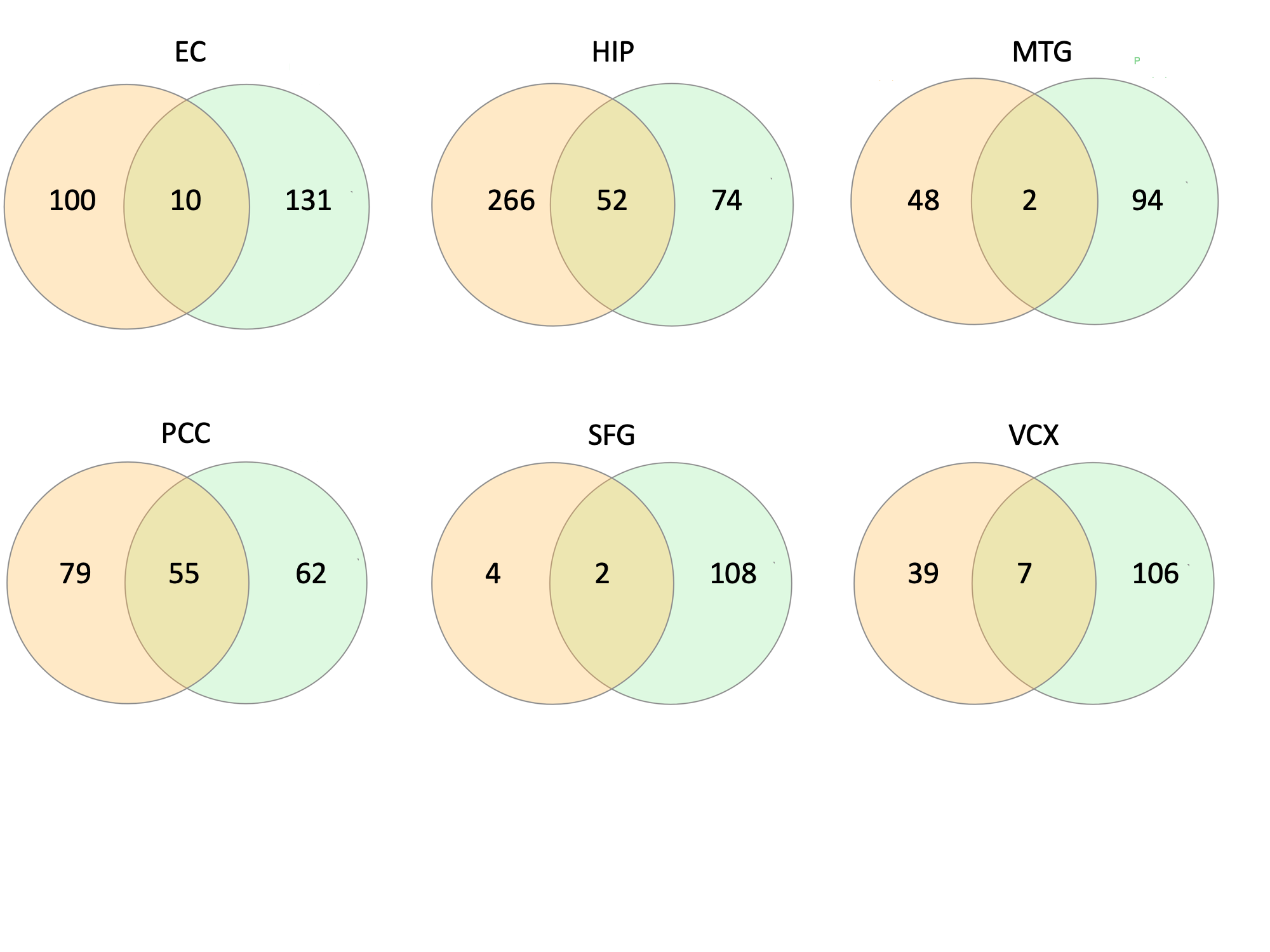

Supplement: S2 Fig — The Venn diagrams were created using http://www.interactivenn.net/. The orange sets represent the SWIM genes whereas the green sets represent the seed genes from Caberlotto et al study. (TIF) [file pone.0222921.s002.tif]

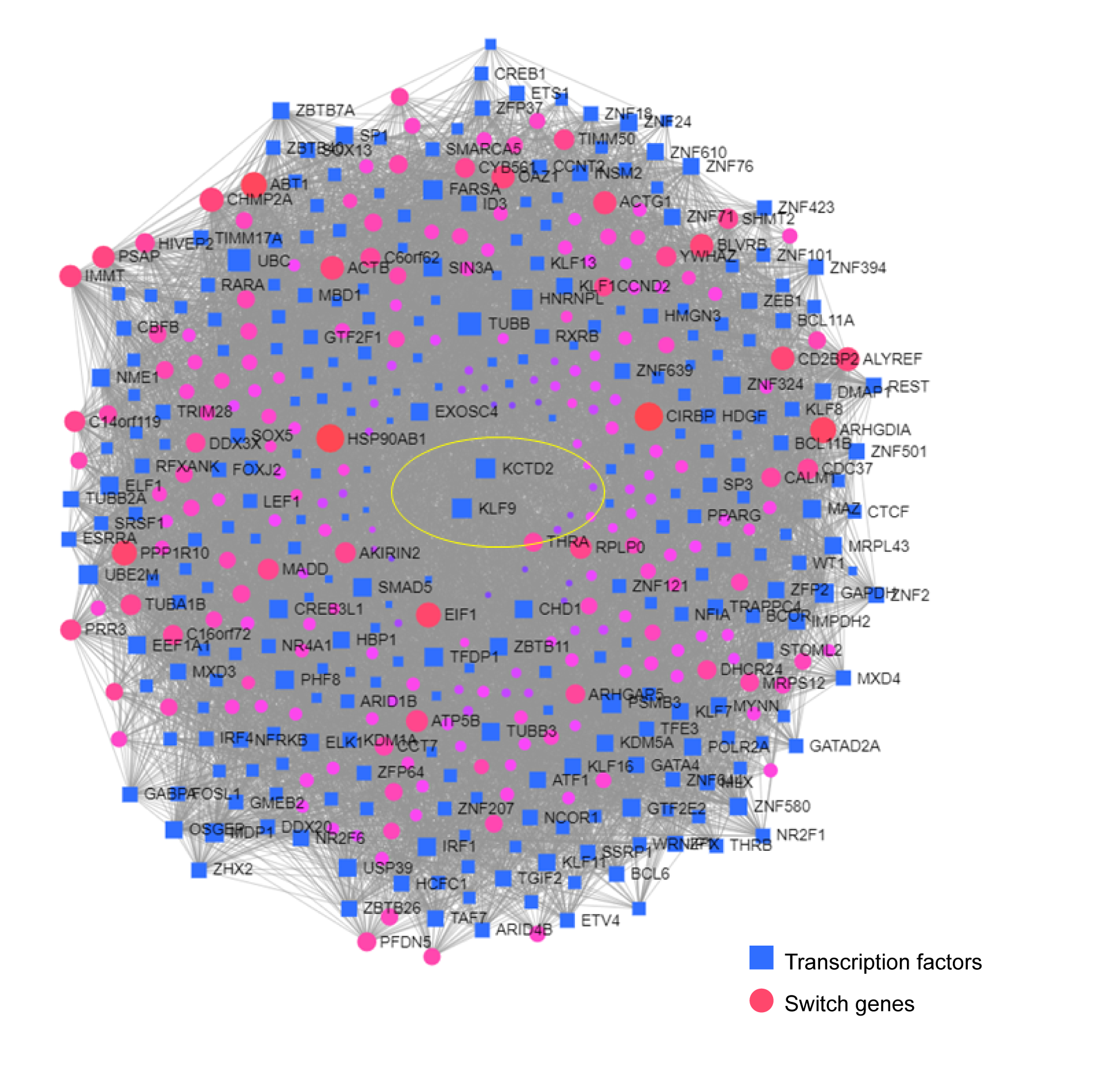

Supplement: S3 Fig — Network analysis of HIP switch genes was performed using NetworkAnalyst. Transcription factor data was derived from the ENCODE ChIP-seq database. Transcription factors (blue rectangles) and switch genes (pink circles) are ranked according to network topology measurements, degree and betweenness centrality. Transcription factors with the highest values of degree and betweenness centrality measurements are enclosed in the yellow oval. Gray lines represent protein-protein interactions. Network analysis was performed on June 2019. (TIF) [file pone.0222921.s003.tif]

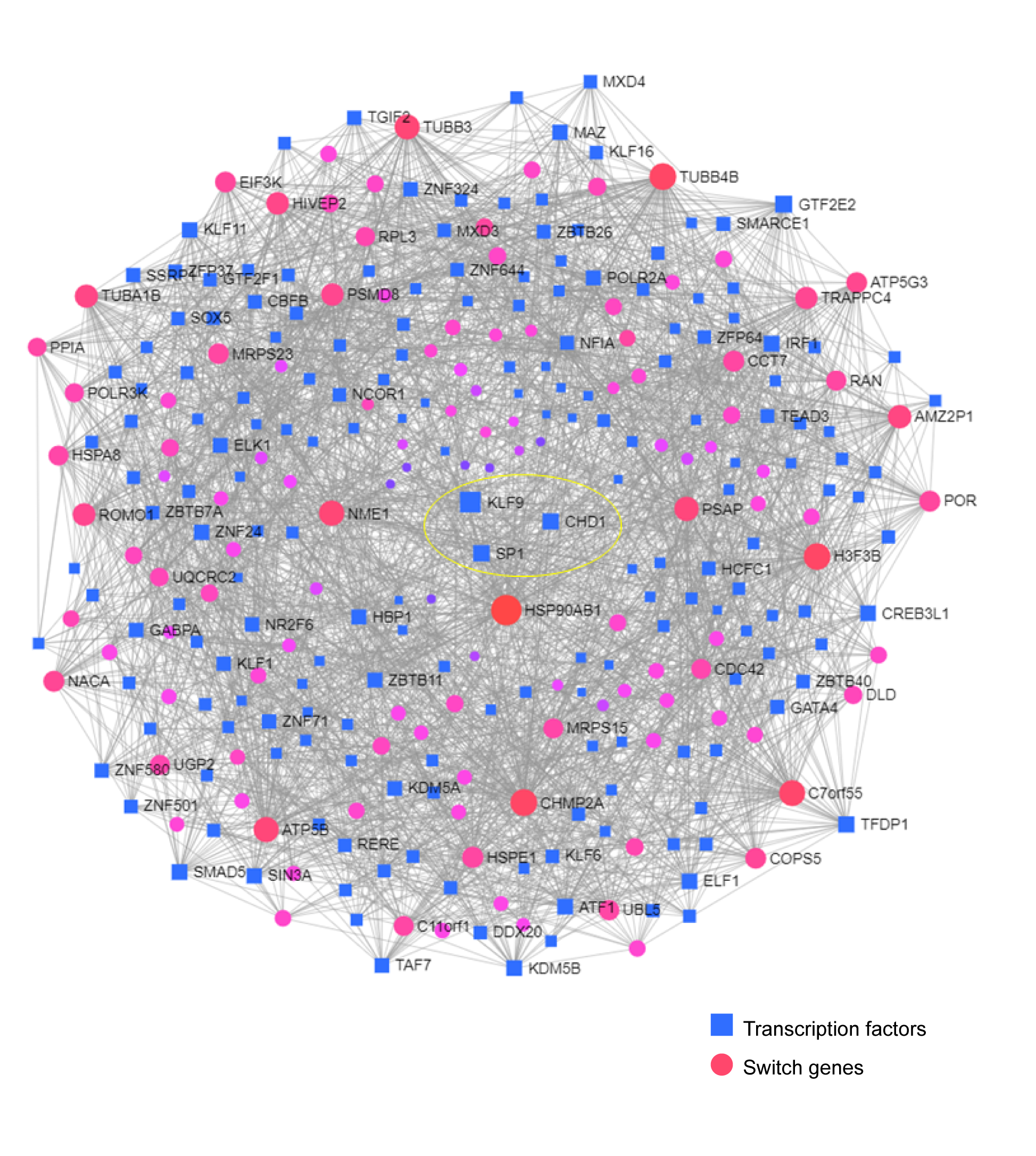

Supplement: S4 Fig — Network analysis of posterior cingulate cortex switch genes was performed using NetworkAnalyst. Transcription factor data was derived from the ENCODE ChIP-seq database. Transcription factors (blue rectangles) and switch genes (pink circles) are ranked according to network topology measurements, degree and betweenness centrality. Transcription factors with the highest values of degree and betweenness centrality measurements are enclosed in the yellow oval. Gray lines represent protein-protein interactions. Network analysis was performed on June 2019. (TIF) [file pone.0222921.s004.tif]
